# Psychiatric Symptoms & Misdiagnosis Of Frontotemporal Dementia: A Case Report

**DOI:** 10.1192/j.eurpsy.2023.1941

**Published:** 2023-07-19

**Authors:** G. Özmen, D. Göverti

**Affiliations:** 1 Erenkoy Training and Research Hospital for Psychiatry and Neurological Diseases Hospital, istanbul, Türkiye

## Abstract

**Introduction:**

Frontotemporal dementia (FTD) is the second most common type of dementia seen between 45-65 years of age and affects the frontal and/or temporal lobes. FTD is clinically characterized by progressively the change in behavior, personality, and language dysfunctions.

Clinical features of FTD include restlessness, disinhibition, apathy, blunted affect, avolition, social withdrawal, impulsiveness, and loss of executive function. Most patients with FTD act as socially inappropriate behaviors, not talking much, compulsive-like acts, poor insight, and psychiatric features including hallucinations and paranoid delusions. According to symptoms, the diagnosis of FTD may be confused with depression, mania, or schizophrenia.

**Objectives:**

In this case report, we wanted to draw attention that FTD should be considered in the differential diagnosis of late-onset psychosis.

**Methods:**

A 53-year-old female, married, uneducated, and not having children patient has applied to our clinic with complaints, that started a year ago, about social withdrawal, activity, decreased sense of purpose, neglecting personal hygiene, not eating well, and acting inappropriately and impulsively.

The patient reported that she was walking out of the house for hours, having profanity speeches, and forgetfulness.

She was admitted to the psychiatry hospital a year ago and discharged with a diagnosis of bipolar disorder.

Brain MRI showed atrophy of frontal and anterior temporal structures bilaterally. PET scan demonstrated left frontal, parietal, and temporal hypo perfusion of the brain.

In our clinical observation, she had apathy, inappropriate jokes, lack of eye contact, flat affect, lack of gesturing when communicating, unable to respond to questions, and visual hallucinations.

**Results:**

Due to the similarity of the clinical resemblance of BPAD and FTD, the diagnosis of FTD can be confusing. In this case, amnesia and sudden onset of the symptoms with rapid destruction may help the diagnosis of FTD.

Psychosis symptoms in our clinical observation also suggested the diagnosis of psychosis. However, its atypical course and early-onset psychosis symptoms brought us closer to organic pathology. Investigations of structural and functional brain imaging may help support the diagnosis.

**Image:**

**Figure:**
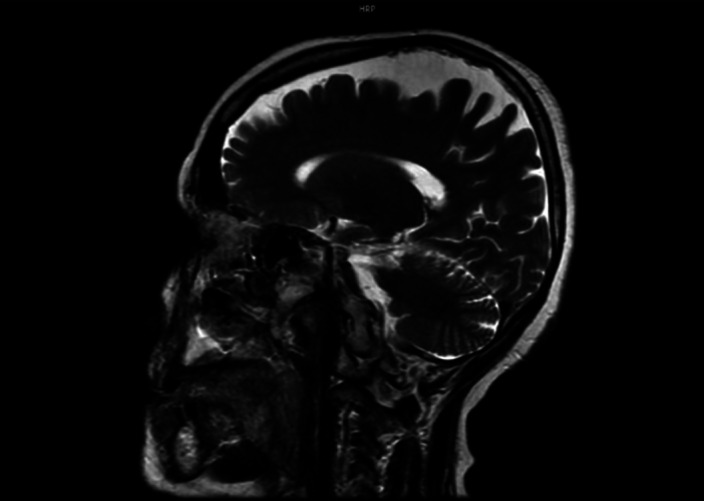

**Image 2:**

**Figure:**
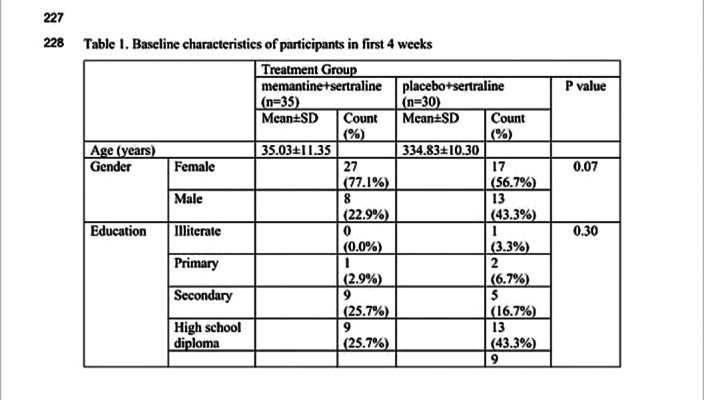

**Conclusions:**

Psychiatric symptoms in FTD may cause misdiagnosis. Organic pathologies should be kept in mind, especially in late and sudden onset symptoms.

**Disclosure of Interest:**

None Declared

